# Meta-Analysis on the Neutrophil-Lymphocyte Ratio in Rectal Cancer Treated With Preoperative Chemoradiotherapy: Prognostic Value of Pre- and Post-Chemoradiotherapy Neutrophil-Lymphocyte Ratio

**DOI:** 10.3389/fonc.2022.778607

**Published:** 2022-02-11

**Authors:** Soo Jin Lee, Kyubo Kim, Hae Jin Park

**Affiliations:** ^1^ Department of Nuclear Medicine, Hanyang University Medical Center, Seoul, South Korea; ^2^ Department of Radiation Oncology, Ewha Womans University College of Medicine, Seoul, South Korea; ^3^ Department of Radiation Oncology, Hanyang University College of Medicine, Seoul, South Korea

**Keywords:** rectal cancer, meta-analysis, neutrophil-lymphocyte ratio, radiotherapy, chemoradiotherapy

## Abstract

**Background:**

To evaluate the prognostic value of neutrophil-lymphocyte ratio (NLR) in rectal cancer patients treated with preoperative chemoradiotherapy (CRT) and curative surgery.

**Methods:**

A comprehensive search of the EMBASE and PubMed databases was performed to screen studies that compared treatment outcomes according to the pre-CRT and/or post-CRT NLR in patients receiving preoperative CRT and curative surgery for locally advanced rectal cancer. Hazard ratios (HRs) for disease-free survival (DFS) and/or overall survival (OS) were extracted, and a random-effects model was used for pooled analysis.

**Results:**

Totally, 22 retrospective studies comprising 6316 patients were included. Preoperative CRT was administered with concurrent chemotherapy (mostly fluoropyrimidine-based regimens). The elevated pre-CRT NLR was significantly associated with an increased risk of recurrence (HR, 1.54; 95% confidence interval [CI], 1.31-1.81) and death (HR, 2.14; 95% CI, 1.61-2.84). Post-CRT NLR was reported in only 3 of 22 studies, and the correlation was not statistically significant for recurrence (HR, 1.44; 95% CI, 0.86-2.41) or death (HR, 2.38; 95% CI, 0.94-6.07).

**Conclusions:**

Elevated pre-CRT NRL, but not post-CRT NRL, is associated with inferior DFS and OS. Further studies are needed to confirm the prognostic value of NLR in rectal cancer patients receiving preoperative CRT.

## Introduction

In locally advanced rectal cancer, preoperative chemoradiotherapy (CRT) has been the standard of care since the landmark study by Sauer et al. (1) For those patients treated with preoperative CRT, several prognostic factors based on tumor characteristics have been reported including ypT classification, ypN classification, tumor regression grade, and neoadjuvant rectal score (2).

Moreover, systemic inflammation-related biomarkers are increasingly applied to predict treatment outcomes. Among these, the neutrophil-lymphocyte ratio (NLR) assay is cost-effective and easy to perform, and its prognostic value has been reported in many malignancies (3).

Recently, Choi et al. (4) evaluated the prognostic impact of pre-RT NLR using 38 datasets including head and neck cancer, rectal cancer, and lung cancer. Among these, the treatment aim was preoperative in 6 of 38 datasets, and all of them were of rectal cancers. The hazard ratio (HR) of pre-RT elevated NLR for overall survival (OS) was 2.45, which was greater in rectal cancer than other primary tumors. However, only 990 rectal cancer patients were analyzed in their study.

Therefore, we conducted this systematic review and meta-analysis to determine the correlation between NLR and treatment outcomes in patients receiving preoperative CRT followed by curative surgery for locally advanced rectal cancer.

## Methods

This systematic review was conducted using structured search terms following the Preferred Reporting Items for Systematic Reviews and Meta-Analyses guidelines (**Supplementary Table 1**)

### Search Strategy

In February 2021, two authors (Lee and Park) performed a comprehensive computer literature search of two databases (EMBASE and PubMed) to identify relevant published studies without a time period limitation. An additional manual search using the reference lists of related literature was also performed. The studies identified from the literature search were evaluated for duplicates; then, full-text articles were independently assessed by two authors (Lee and Park) to determine the eligibility of each article. Studies irrelevant to our research questions were eliminated.

The following search criteria were used: (‘neutrophil lymphocyte ratio’ OR ‘neutrophil-to-lymphocyte ratio’ OR ‘neutrophil-lymphocyte ratio” OR NLR) *AND* (‘rectal neoplasms’ OR ‘rectal neoplasm’ OR ‘rectal tumor’ OR ‘rectal cancer’). All searches were limited to full-text articles and human studies written in English. All patients and both prospective and retrospective studies were included.

### Inclusion and Exclusion Criteria

Two authors (Kim and Park) independently reviewed the screened studies, and any discrepancies were resolved by discussions. Studies in our meta-analysis were included only if they mentioned all of the following: 1) histologically confirmed rectal cancer; 2) preoperative CRT followed by curative resection, 3) NLR measured before and/or after CRT; 4) association of NLR with OS and/or disease-free survival (DFS); 5) HR with 95% confidence interval (CI), or sufficient data to estimate HR and standard error (SE). The studies were excluded if the HR and its SE could not be extracted from the reported data. Moreover, studies of poor quality published in journals without an impact factor were excluded by discussion. When the data considered were published in more than one article, only the first related article was included.

### Data Extraction

Two reviewers (Kim and Park) independently extracted data from each article and record them on a standardized form. Any disagreement in data extraction was resolved by discussion. The following data were extracted from each article: (1) first author’s name, year of publication, median age of patients, number of included patients, study design (prospective or retrospective), and follow-up periods; (2) detailed information on treatment, TNM stage, and NLR cut-off value; (3) raw data as well as HR for DFS or OS before and after CRT. When the data were presented as a figure, data extraction was performed using Engauge Digitizer software (version 10.4, http://markummitchell.github.io/engauge-digitizer/). If included studies presented HRs by both univariate and multivariate analysis, the former one was used for pooling data across studies.

### Assessment of Risk of Bias and Quality Assessment

We generated funnel plots to assess possible publication bias. The asymmetricity of plots was tested using trim and fill methods, and the pooled risk estimates were recalculated with the addition of those missing studies.

Quality assessment of the studies was based on the Newcastle-Ottawa scale (NOS); an NOS score of ≥ 6 indicated high-quality studies. Consensus was reached by discussion when disparity occurred. After quality assessment, only high-quality studies were included in our analysis (**Supplementary Table 2**)

### Statistical Analysis

All analyses and corresponding plots were performed using the statistical software R version 3.6.1 (R Foundation for Statistical Computing) (5) using the “meta” (6) and “metaphor” (7) package. A random-effects model was constructed for all included studies to analyze DFS and OS associated with NLR before and after CRT. Heterogeneity was assessed by the likelihood ratio *I^2^* index, which is considered high when > 50%. Subgroup analysis was performed to determine whether some individual studies explained heterogeneity and to assess the consistency of the results.

## Results

### Study Selection and Characteristics

**Figure 1:**
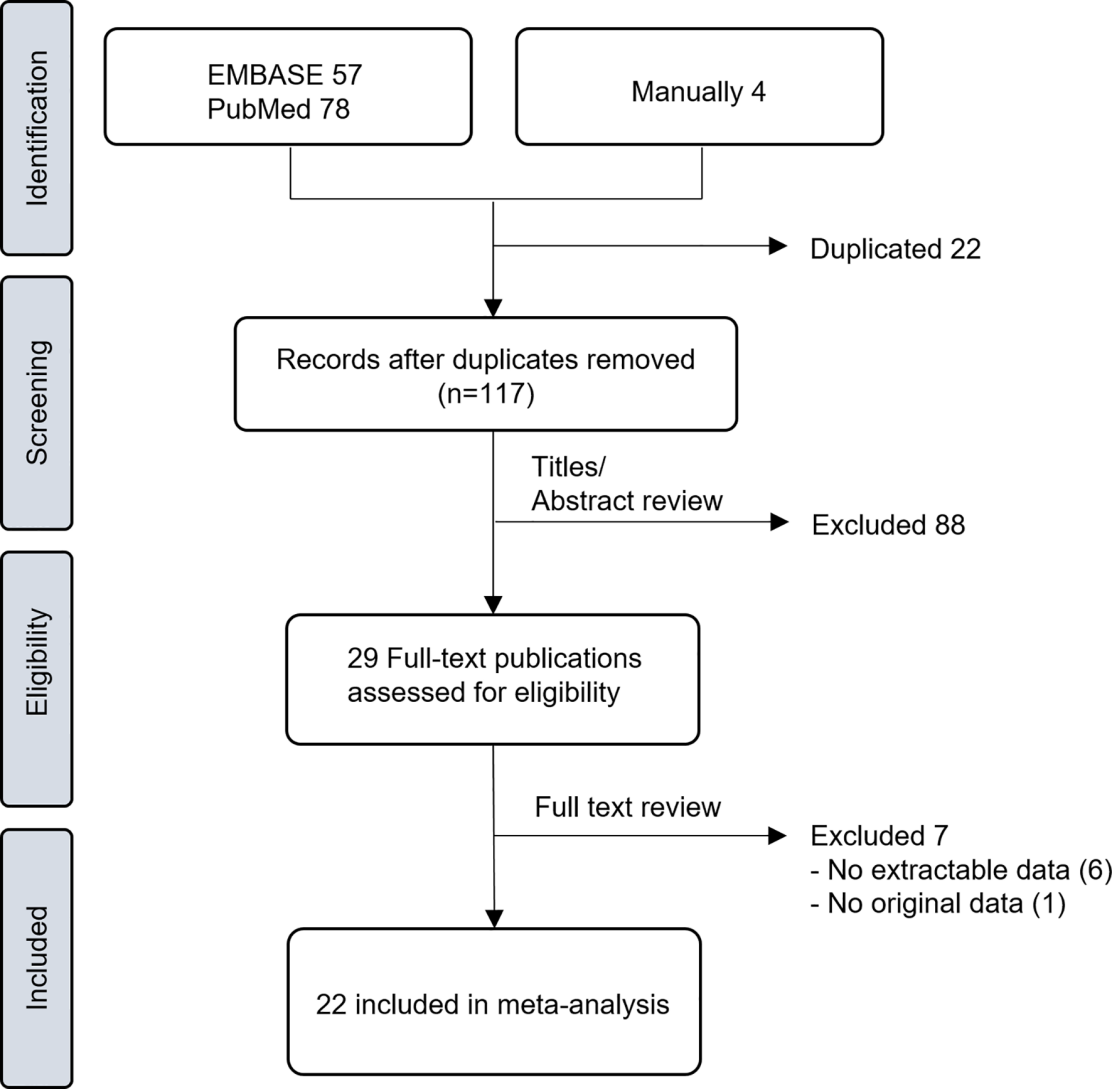
Flow diagram of study selection.

A total of 117 studies (57 from EMBASE, 78 from PubMed, and 4 from manual searches) were screened after the removal of duplicates (**Figure 1**). Based on the titles and abstracts, 29 studies were judged as potentially relevant and evaluated in more detail. After reviewing the full text, 7 studies (8–14) were excluded for not meeting the eligibility criteria. The remaining 22 studies (15–36) comprising 6316 rectal cancer patients were included in this meta-analysis. Important characteristics of these studies are presented in **Table 1**. All studies were retrospective observational studies. Twenty studies reported the association between pre-CRT NLR and treatment outcomes, and 3 studies (27, 31, 33) reported the association between both pre-CRT and post-CRT NLR and treatment outcomes. The sample size of individual studies was 48–1527, and the mean age of patients was 51–66 years. Most studies adopted long-course CRT, mostly concurrent with fluoropyrimidine-based regimens. Two-thirds utilized adjuvant chemotherapy, but one-third did not provide information on this. The cut-off value of NLR by various methods was 1.7–5 for DFS and OS.

**Table 1:** Details of the included studies.

First author	Year of publication	Country	Number of patients	Median age (years)	Median follow-up (months)	Pre-CRT stage	Treatments, RT	Treatments, concurrent CTx	Treatments, adjuvant CTx	Cut-off value of NLR	Cut-off method	Outcome	NOS
Carruthers R (15)	2012	UK	115	63.8	37.1	cT3-4	45 Gy/25 fx	5-FU + folic acid	NA	5	NA	DFS, OS	7
Toiyama Y (16)	2013	Japan	84	64.5	56	I, II, III	20 Gy/4 fx (57%), 45 Gy/25 fx (43%)	5-FU + tegafur/uracil	5-FU-based	3	NA	DFS, OS	7
Shen L (18)	2014	China	199	55	31	II, III	median 50 Gy, 1.8-2.0 Gy/fx	5-FU-based	92.5%, regimen NA	2.8	ROC analysis	DFS, OS	7
Kim IY (17)	2014	Korea	102	NA	39	II, III	50.4 Gy/28 fx	5-FU + leucovorin	5-FU + leucovorin	3	NA	DFS, OS	7
Nagasaki T (19)	2015	Japan	201	61	51.2	II, III	45-50 Gy, 1.8-2.0 Gy/fx	5-FU-based	45.3%, regimen NA	3	ROC analysis	DFS, OS	7
Hodek M (20)	2016	Czech Republic	173	62.8	35	locally advanced	50.4 Gy/28 fx	5-FU	83.4%, regimen NA	2.8	log-rank statistics	DFS, OS	7
Jung SW (21)	2017	Korea	984	59	48	II, III	50.4-55.4 Gy/28 fx	5-FU + leucovorin or Capcitabine	NA	1.7	ROC analysis	DFS	7
Zhao J (23)	2017	China	100	60.5	45.5	II, III	50-55 Gy/25 fx	Capcitabine-based	NA	2.25	ROC analysis	OS	7
Shen J (22)	2017	China	202	51	45	II, III	45-55 Gy, long-course	5-FU-based (FOLFIRI or FOLFOX)	53.5%, regimen NA	3	ROC analysis	DFS, OS	7
Vallard A (24)	2018	France	257	66	46.1	I, II, III	median EQD2 49.2 Gy, HypoFx 7.8% included	5-FU or FOLFOX (4.6% not done)	38.8%, FOLFOX or 5-FU	2.8	log-rank statistics	DFS, OS	7
Ward WH (25)	2018	USA	146	58.6	NA	II, III	median 50.4 Gy, 1.8-2.0 Gy/fx	5-FU or Capcitabine or multi-agent	NA	4.47	maximally selected rank statistics	DFS, OS	6
Kim TG (29)	2019	Korea	176	57	75	II, III	44-45 Gy, 1.8-2.0 Gy/fx	5-FU or Capcitabine	100%, regimen NA	2	ROC analysis	DFS, OS	8
Dudani S (28)	2019	Canada	1527	62	71	II, III	median 50 Gy, long-course	5-FU or Capcitabine	83%, 5-FU-based	4	NA	DFS, OS	8
Braun LH (26)	2019	Germany	220	65.5	67	II, III	long-course	5-FU-based	55.5%, regimen NA	4.06	ROC analysis	DFS	8
Cha YJ (27)	2019	Korea	131	59	73.3	II, III	mean 50.4 Gy/28 fx	5-FU or Capcitabine	85.5%, mainly 5-FU or Capcitabine	3	NA	DFS, OS	8
Zhang X (30)	2019	China	76	NA (74%, <60)	23	II, III	50 Gy/25 fx or 25 Gy/5 fx	5-FU-based for long-course RT	NA	2	Cutoff Finder software	OS	6
Sun Y (34)	2020	China	317	55.5	54	II, III	50.4 Gy/28 fx	capecitabine + oxaliplatin or FOLFOX	capecitabine + oxaliplatin or FOLFOX	2.9	X-tile analysis	DFS, OS	7
Ke T (32)	2020	Taiwan	184	63.17	72.7	I, II, III	45-50 Gy/25-28 fx	5-FU-based	100%, FOLFOX or 5-FU/leucovorin or capcitabine	3.5	mean value	DFS, OS	8
Lee JH (33)	2020	Korea	549	61	NA	I, II, III	median 50.4 Gy, 1.8-2.0 Gy/fx	5-FU or Capcitabine	NA	2	median value	DFS, OS	6
Zhang Y (35)	2020	China	472	56	NA	II, III	50.4 Gy/28 fx	done, regimen NA	done, regimen NA	2.3	X-tile analysis	DFS, OS	6
Ishikawa D (31)	2020	Japan	48	66	36 for DFS, 60 for OS	I, II, III	40 Gy/20 fx	tegafur-gimeracil-oteracil or tegafur-uracil or 5-FU	NA	2.45	median value	DFS, OS	6
Ergen SA (36)	2021	Turkey	53	55	43	II, III	50.4 Gy/28 fx	Capcitabine	43.4%, regimen NA	2.49	ROC analysis	OS	6

CRT, chemoradiotherapy; RT, radiotherapy; CTx, chemotherapy; NLR, neutrophil-lymphocyte ratio; NOS, Newcastle-Ottawa Quality Assessment Scale; NA, not available; DFS, disease-free survival; OS, overall survival; ROC, receiver operating characteristic.

### Meta-Analysis Results

**Figure 2:**
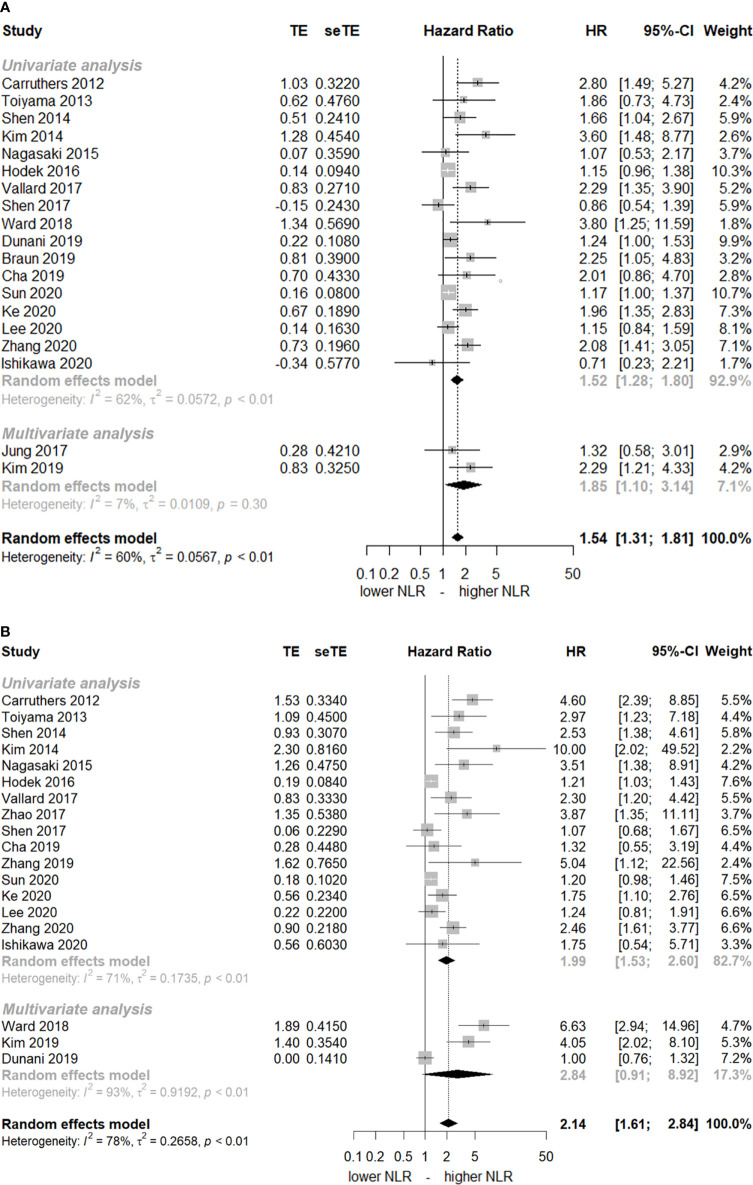
Forest plot of hazard ratios (HR) for an elevated neutrophil-lymphocyte ratio (NLR) before chemoradiotherapy. **(A)** disease-free-survival and **(B)** overall survival. The squares represent HR for each study and their size represents the weight of the study in the meta-analysis. The horizontal lines crossing the squares represent the 95% confidence intervals (CI).

The HRs of DFS and OS with pre-CRT NLR were 1.54 (95% CI, 1.31–1.81) and 2.14 (95% CI, 1.61–2.84), respectively (**Figure 2**). Substantial heterogeneity was noted (*I ^2 =^* 60% and 78%, respectively). The elevated pre-CRT NLR was strongly associated with worse treatment outcomes in patients with rectal cancer. Funnel tests using the trim and fill methods were conducted for publication bias assessment; **Supplementary Figure 1** shows asymmetry, indicating publication bias in both DFS and OS. After the application of the trim and fill methods, the adjusted pooled analysis still presented increased HRs for both DFS and OS (adjusted HR of DFS = 1.31, 95% CI, 1.10–1.55; adjusted HR of OS = 1.36, 95% CI, 0.96–1.93).

**Figure 3:**
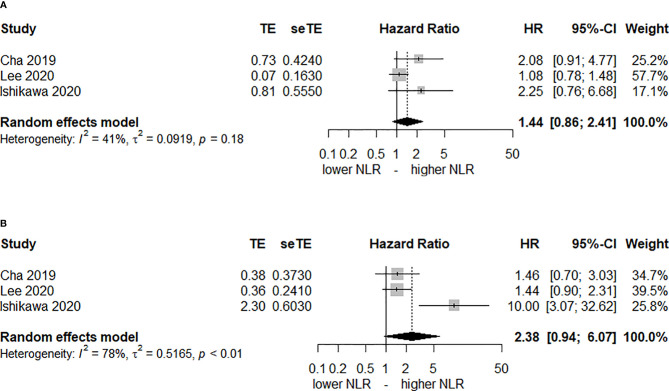
Forest plot of hazard ratios (HR) for an elevated neutrophil-lymphocyte ratio (NLR) after chemoradiotherapy. **(A)** disease-free-survival and **(B)** overall survival.

Only 3 studies (27, 31, 33) reported the post-CRT NLR as well. **Figure 3** shows that elevated post-CRT NLR is not associated with DFS or OS. The HRs of DFS and OS were 1.44 (95% CI, 0.86–2.41, *I^2^* = 41%) and 2.38 (95% CI, 0.94–6.07, *I^2^* = 45%), respectively.

## Discussion

In this meta-analysis, elevated pre-CRT NLR was associated with 1.5- and 2-fold higher risks of relapse and death, respectively, in patients treated with preoperative CRT and curative surgery for locally advanced rectal cancer.

An elevated NLR could result from either neutrophilia or lymphopenia, both of which are reported to be associated with poor prognosis in many malignancies (37). Neutrophils are known to release inflammatory cytokines such as nuclear factor kappa B as well as reactive oxygen and nitrogen species, and they can promote angiogenesis and tumor growth. Neutrophilia can predict inferior survival outcomes in rectal, anal, and cervical cancers (38–40). Lymphopenia inversely correlates with the number of tumor-infiltrating lymphocytes, and therefore, it might suggest impaired antitumoral immunity, which in turn could promote tumor progression. Given that lymphocytes are highly radiosensitive, and treatment-related lymphopenia is often reported, the utility of using post-RT lymphopenia as a prognostic factor in various tumors has been suggested (41). Although not as often as post-RT lymphopenia, pre-RT lymphopenia is also reported to correlate with treatment outcomes. Li et al. (42) observed that pre-RT lymphopenia is associated with poorer survival after stereotactic radiosurgery in lung cancer patients with brain metastases. Although the response to preoperative CRT was not analyzed in our study, Hodek et al. (20) and Kim et al. (17) noted that the NLR level is correlated with pathologic tumor response as well as survival. Choi et al. (4) suggested that hypoxia, which is well known to decrease response to radiation therapy, could be driving this relationship. They speculated that tumor growth and necrosis create a hypoxic environment, which triggers an inflammatory response by modulating cytokine expression.

Recently, Hamid et al. (43) conducted a meta-analysis regarding the prognostic impact of NLR in rectal cancer patients undergoing curative resection and noted that pre-treatment NLR is significantly correlated with DFS and OS. However, they analyzed mixed populations treated with surgery alone or preoperative CRT followed by surgery. Our study included only patients receiving preoperative CRT before surgery, and pre-CRT NLR significantly correlated with survival. Meanwhile, a Canadian multicenter study (28) with the largest effect size reported that an elevated pre-CRT NLR level is not associated with OS on both univariate and multivariate analyses. When the patient and tumor characteristics were compared according to the pre-CRT NLR level, a higher pre-CRT NLR was associated with older age, poor performance status, and lower hemoglobin level, of which age and performance status were significant prognostic factors affecting OS on multivariate analysis. Therefore, further studies are needed to confirm the prognostic value of NLR after adjusting for potential confounding factors.

Total neoadjuvant treatment (TNT) is an emerging issue in the management of locally advanced rectal cancer, and early results from randomized controlled trials are promising (44, 45). However, the impact of NLR in the setting of TNT has not yet been established. Recently, Roy et al. (46) noted that baseline NLR level is associated with DFS and OS on univariate analysis but not on multivariate analysis in patients receiving short-course RT (25 Gy in 5 fractions) followed by chemotherapy and delayed surgery. It was not clear whether the lack of significant correlation between NLR and the outcomes in the aforementioned study was due to the hypofractionated RT or the treatment sequencing. However, in the TNT setting employing upfront CRT, post-CRT NLR might give additional information regarding the best candidate requiring post-CRT chemotherapy prior to surgery. Treatment-related lymphopenia is reported to be common in rectal cancer patients undergoing neoadjuvant CRT, and its association with a poor prognosis has also been observed (47, 48). In the present study, only 3 articles reported the impact of post-CRT NLR, but the correlation was not statistically significant, and only unadjusted HRs were reported. Further research is warranted for estimating the implication of post-CRT NLR in the context of TNT. Moreover, such post-CRT biomarkers might facilitate the selection of potential candidates of “watch-and-wait” strategy.

There are some shortcomings in this study. First, there were only 3 studies to report adjusted HRs for pre-CRT NLR. There could be an underlying correlation between pre-CRT NLR and baseline patient and/or tumor characteristics, and therefore, the impact of NLR might disappear on multivariate analysis after adjusting for potential covariates. Second, patient-, tumor-, and treatment-related heterogeneities, as well as different cut-offs for the NLR level applied in the included studies, could have influenced the results. Third, the inclusion of different treatments might affect the results of our study: 3 studies employed short-course radiotherapy in part and another 3 studies also employed oxaliplatin-based regimen as the concomitant chemotherapy in part. Lastly, we did not analyze the effect of pre- and post-CRT NLR on pathologic tumor response because of the lack of reported HRs in included studies. Another meta-analysis focused on this important topic is warranted in the future.

In conclusion, elevated pre-CRT NLR is associated with inferior DFS and OS in rectal cancer, but post-CRT NRL is not. Further studies are needed to confirm the prognostic value of NLR in rectal cancer patients receiving preoperative CRT.

## Data Availability Statement

The original contributions presented in the study are included in the article/**Supplementary Material**. Further inquiries can be directed to the corresponding authors.

## Author Contributions

KK and HP designed the study and collected the data. SL performed the statistical analysis of the collected data. All three authors wrote the manuscript. All authors contributed to the article and approved the submitted version.

## Funding

This work was carried out with the support of the research fund of Hanyang University (HY-2020).

## Conflict of Interest

The authors declare that the research was conducted in the absence of any commercial or financial relationships that could be construed as a potential conflict of interest.

## Publisher’s Note

All claims expressed in this article are solely those of the authors and do not necessarily represent those of their affiliated organizations, or those of the publisher, the editors and the reviewers. Any product that may be evaluated in this article, or claim that may be made by its manufacturer, is not guaranteed or endorsed by the publisher.

## Supplementary Material

Supplementary Figure 1 Funnel plots with trim-and-fill. **(A)** disease-free-survival and **(B)** overall survival before chemoradiotherapy.
