# Supplementary figures and images for: Meta-Analysis on the Neutrophil-Lymphocyte Ratio in Rectal Cancer Treated With Preoperative Chemoradiotherapy: Prognostic Value of Pre- and Post-Chemoradiotherapy Neutrophil-Lymphocyte Ratio
Source: Front Oncol. 2022 Feb 11;12:778607. doi: 10.3389/fonc.2022.778607 (PMC8873579; doi:10.3389/fonc.2022.778607)

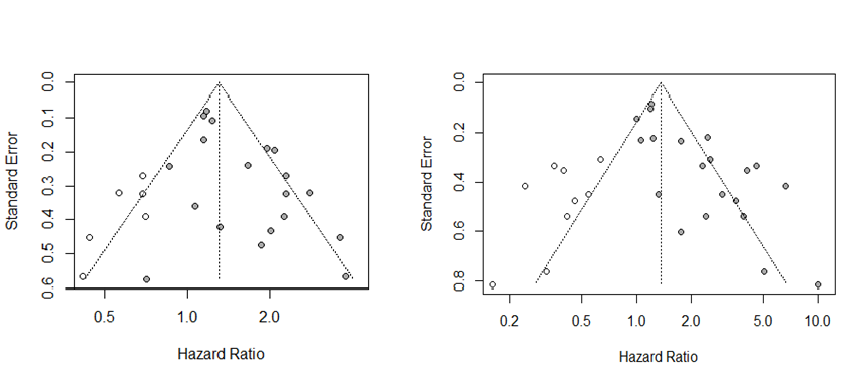

Supplement: Supplementary Figure 1 — Funnel plots with trim-and-fill. (A) disease-free-survival and (B) overall survival before chemoradiotherapy. [file Image_1.tif]
